# Dysregulation of serum miR-361-5p serves as a biomarker to predict disease onset and short-term prognosis in acute coronary syndrome patients

**DOI:** 10.1186/s12872-021-01891-0

**Published:** 2021-02-05

**Authors:** Wenqing Zhang, Guannan Chang, Liya Cao, Gang Ding

**Affiliations:** 1 Department of Geriatrics, Yidu Central Hospital of Weifang, WeifangShandong, 262500 China; 2 Department of Cardiology, Yidu Central Hospital of Weifang, No. 4138, Linglongshan South Road, Qingzhou, Weifang, 262500 Shandong China; 3 Department of Gynecology Ward II, Yidu Central Hospital of Weifang, Weifang, 262500 Shandong China; 4 Department of Science and Education, Yidu Central Hospital of Weifang, Weifang, 262500 Shandong China

**Keywords:** MicroRNA-361-5p, Acute coronary syndrome, Diagnosis, Prognosis, Endothelial dysfunction

## Abstract

**Background:**

Serum microRNAs (miRNAs) have been used as novel biomarkers for various diseases, including acute coronary syndrome (ACS). This study aimed to investigate the expression and clinical significance of microRNA-361-5p (miR-361-5p) in patients with ACS.

**Methods:**

This study included 118 ACS patients, 78 patients with stable coronary heart disease (SCHD) and 66 healthy controls. MiR-361-5p expression was measured by qRT-PCR. The diagnostic value of miR-361-5p was evaluated by the ROC analysis. A 30-day follow-up was performed for the patients from hospitalization, and Kaplan–Meier curves and logistics analysis were used to evaluate the ability of miR-361-5p to predict the occurrence of major adverse cardiac events (MACE). ELISA kits were used to detect the levels of endothelial dysfunction (ED) markers, including vascular cell adhesion molecule 1 (VCAM-1), intercellular adhesion molecule 1 (ICAM-1) and E-selectin.

**Results:**

The expression of miR-361-5p was significantly increased in patients with SCHD and ACS, and positively correlated with Gensini scores. Serum miR-361-5p expression had a high diagnostic accuracy for distinguishing ACS from health controls and SCHD patients. ACS patients with high expression of miR-361-5p had a higher probability of developing MACE. MiR-361-5p expression was an independent risk factor for the occurrence of MACE in ACS patients, and was positively correlated with the levels of VCAM-1, ICAM-1 and E-selectin.

**Conclusion:**

All data indicated that miR-361-5p expression was significantly increased in ACS patients. Aberrant miR-361-5p expression in ACS might be a candidate biomarker for ACS diagnosis and the the prediction of MACE onset.

## Introduction

Coronary heart disease (CHD) is a major cardiovascular disease that threatens human life, health and safety. It can be classified into stable CHD (SCHD) and acute coronary syndrome (ACS). ACS is the most severe medical emergency caused by myocardial ischemia following the formation of unstable coronary atherosclerotic plaques, and its clinical manifestations include acute myocardial infarction (AMI) and unstable angina pectoris (UA) [1]. Currently, the understanding of the pathological mechanism of ACS remains limited, leading to very few biomarkers for the early screening and prognosis of ACS. It has been found that endothelial dysfunction (ED) plays a key role in the initiation, progression and the rupture of atherosclerotic thrombotic plaques [2]. In addition, the pathological mechanisms of ACS also involve coronary atherosclerotic plaque rupture and thrombosis. Therefore, ED plays an important role in the pathological mechanisms of ACS. Some studies have pointed out that some reported molecular markers can reflect the occurrence of ED, such as vascular cell adhesion molecule 1 (VCAM-1), intercellular adhesion molecule 1 (ICAM-1) and E-selectin [3]. When endothelial cells are damaged, these molecules are rapidly released, thereby reflecting the damage of endothelial cell. Therefore, exploring the correlation between endothelial function-related molecules and ACS disease progression is expected to find more potential biomarkers and therapeutic targets for ACS disease.

MicroRNAs (miRNAs) are a class of small (about 22 nucleotides), noncoding single stranded RNAs that regulate gene expression by binding to the 3′-untranslated region (3′-UTR) of target mRNAs for mRNA degradation or translation suppression [4]. A study has demonstrated that miRNAs can be used as potential biomarkers for the diagnosis and prognosis of patients with cardiovascular diseases, including ACS [5]. At present, a large number of miRNAs, such as miR-29b [6] and miR-210 [7], have been found to regulate the biological function of endothelial cells, and are thereby closely associated with endothelial function. In addition, early studies reported that miR-361-5p has anti-angiogenic effects through regulating endothelial cell activity [8]. Moreover, a significant increase in the expression of miR-361-5p has been found in CHD patients, it can participant in the regulation of endothelial cell activity and function, and was closely related to the occurrence and development of CHD disease by regulating vascular endothelial growth factor (VEGF) [9]. However, the clinical value of miR-361-5p in ACS patients remains unknown.

This study aimed to analyze the expression levels of miR-361-5p in ACS patients, evaluate the clinical significance of miR-361-5p in screening ACS patients and its application value in predicting short-term clinical prognosis in ACS patients. The findings may provide novel ideas for the development of ACS biomarkers.

## Material and methods

### Study population

The present study included ACS patients (n = 118), SCHD patients (n = 78) and healthy controls (n = 66) admitted to the Yidu Central Hospital of Weifang from 2018 to 2019. The diagnosis of ACS was performed according to established international diagnostic criteria for ACS [10–12]. The diagnosis of AMI was defined as ischemic chest pain lasting > 30 min, cardiac biomarker increase [cardiac troponin I (cTnI) > 0.06 ng/mL and/or creatine kinase MB (CK-MB) > 16 IU/L], and electrocardiogram (ECG) findings of a new ST-segment shift in two or more contiguous leads. According to the presence or absence of ST segment elevation in the ECG, AMI was classified into non-ST-segment elevation myocardial infarction (NSTEMI) and ST-segment elevation myocardial infarction (STEMI). UA was defined as patients with a clinical history and ECG consistent with ACS without an increase in cardiac biomarkers. Patients with SCHD were confirmed by ECG and coronary artery angiography. Patients in healthy controls had no coronary stenosis as confirmed by coronary artery angiography. All subjects with autoimmune diseases, acute or chronic infectious diseases, severe liver and kidney dysfunction or malignant tumors were excluded. This clinical study was approved by the Ethics Committee of the Yidu Central Hospital of Weifang.

### Sample collection and storage

Blood samples were collected from SCHD patients and healthy controls before breakfast and from ACS patients within 3 h of symptom onset. Serum samples were isolated from blood by centrifugation at 3000*g* for 10 min at 4 °C, and then were stored at -80 °C for further analyses. Each participant signed an informed consent before sampling.

### Data collection

The baseline characteristics of the participants were recorded at admission, including age, gender, body mass index (BMI), total cholesterol (TC), triglyceride (TG), high-density lipoprotein cholesterol (HDL-C), low-density lipoprotein cholesterol (LDL-C), hypertension, diabetes, white blood cell (WBC), cTnI and Gensini score.

### Calculation of Gensini scores and evaluation of the ED

To assess the disease severity in ACS patients, the Gensini score was calculated according to the degree of coronary stenosis [13]. ELISA kits were used to evaluate the levels of ED markers VCAM-1 (Cat no. #DVC00), ICAM-1 (Cat no. #DCD540) and E-selectin (Cat no. #DSLE00) (R & D System, MN, USA) to reflect the degree of ED in ACS patients.

### Short-term follow up and study endpoints

Patients were followed up from hospitalization to 30 days by face-to-face interview or telephone interview. The primary study endpoint was the occurrence of major adverse cardiac events (MACE), which were defined as a composite of death, heart failure, unstable angina and malignant arrhythmias. These subjects were followed until the occurrence of event, or until 30 days of hospitalization if there were no events. To avoid multiple counts of patients with more than one event, each patient contributed only once to the endpoint. Endpoints were determined by face-to-face interviews or by telephone interviews from hospitalization to 30 days.

### RNA extraction and quantitative real-time PCR (qRT-PCR)

Total RNA was extracted from serum using TRIzol Reagent (Invitrogen, Carlsbad, CA, USA). The purity and concentration of RNA were evaluated using a NanoDrop 2000 (Thermo Fisher, Scientific, Inc.). A PrimeScript RT reagent kit (TaKaRa, Japan) was used to synthesize cDNA from the obtained RNA.

Serum miR-361-5p expression was examined using qRT-PCR, which was carried out using SYBR Green I Master Mix kit (Invitrogen, Carlsbad, CA, USA) and 7300 Real-Time PCR System (Applied Biosystems, USA). All procedures were performed according to the manufacturer’s instructions. The miR-361-5p expression was normalized to U6 and calculated using the 2^−ΔΔCt^ method.

### Statistical analysis

All statistical analyses were performed using SPSS 21.0 software (SPSS, Inc., Chicago, USA) and GraphPad Prism 7.0 software (Inc., Chicago, USA). All data were presented as mean ± standard deviation (SD). Comparisons between measurement data were performed using student’s t test or one-way ANOVA. Chi-square test was used to compare the counting information. Receiver operating characteristic (ROC) curves were plotted to assess the diagnostic value of miR-361-5p. The Kaplan–Meier method was used to analyze the correlation between miR-361-5p expression and the occurrence of MACE, and multivariate logistic analysis was used to assess whether miR-361-5p is an independent risk factor for MACE occurrence in ACS patients. Correlation analysis was performed using Pearson’s correlation coefficient. A *P* < 0.05 indicated statistically significant.

## Results

### Baseline characteristics of the study population

The baseline characteristics of ACS patients, SCHD patients and healthy controls were summarized in Table 1. There were no significant differences in age, gender, BMI, TG, hypertension and diabetes among healthy controls, SCHD patients, and ACS patients. Compared with healthy controls, ACS patients exhibited significantly increased TC, LDL-C, WBC and cTnI, and significantly decreased HDL-C (all *P* < 0.05). In addition, compared with the SCHD patients, ACS patients exhibited significantly increased TC, WBC, cTnI and Gensini score (all *P* < 0.05). Moreover, compared with the healthy controls, decrease in HDL-C and increase in WBC and cTnI were found in the SCHD patients (all *P* < 0.05).

**Table 1:** Baseline characteristics of the study population

Features	Total individuals (n = 262)	Healthy controls (n = 66)	SCHD patients (n = 78)	ACS patients (n = 118)	*P*_1_ value	*P*_2_ value	*P*_3_ value
Age (years)	65.0 ± 9.7	65.0 ± 11.2	63.8 ± 7.9	65.6 ± 9.7	0.759	0.912	0.430
Gender, male (%)	157 (59.9%)	40 (60.6)	49 (62.8)	68 (57.6)	0.785	0.694	0.468
BMI (kg/m^2^)	25.0 ± 2.7	24.7 ± 2.3	25.4 ± 3.2	25.0 ± 2.7	0.324	0.792	0.603
TC (mM)	4.7 ± 0.8	4.6 ± 0.5	4.5 ± 0.6	5.0 ± 0.8	0.935	0.006	0.001
TG (mM)	1.8 ± 0.6	1.6 ± 0.6	1.8 ± 0.5	1.8 ± 0.6	0.512	0.087	0.594
HDL-C (mM)	1.2 ± 0.3	1.5 ± 0.2	1.2 ± 0.2	1.2 ± 0.3	< 0.001	< 0.001	0.979
LDL-C (mM)	2.7 ± 0.6	2.5 ± 0.7	2.6 ± 0.4	2.8 ± 0.6	0.247	0.002	0.173
Hypertension (%)	85 (32.4%)	19 (28.8)	24 (30.8)	42 (35.6)	0.796	0.347	0.484
Diabetes (%)	81 (30.9%)	17 (25.8)	25 (32.1)	39 (33.1)	0.408	0.302	0.884
WBC (× 10^9^/L)	8.0 ± 2.6	6.3 ± 1.4	7.6 ± 1.8	9.1 ± 2.6	0.004	< 0.001	< 0.001
cTnI (ng/mL)	0.8 ± 0.9	0.09 ± 0.1	0.2 ± 0.2	1.6 ± 1.2	< 0.001	< 0.001	< 0.001
Gensini score	–	–	47.5 ± 19.5	58.6 ± 24.4	–	–	0.002

*P*_1_: SCHD patients vs. healthy controls; *P*_2_: ACS patients *vs.* healthy controls; *P*_3_: ACS patients vs. SCHD patients

*SCHD* stable coronary heart disease, *ACS* acute coronary syndrome, *BMI* body mass index, *TC* total cholesterol, *TG* triglyceride, *HDL-C* high-density lipoprotein cholesterol, *LDL-C* low-density lipoprotein cholesterol, *WBC* white blood cell, *cTnI* cardiac troponin I

### Expression of miR-361-5p and its correlation with Gensini scores in ACS patients

**Fig. 1:**
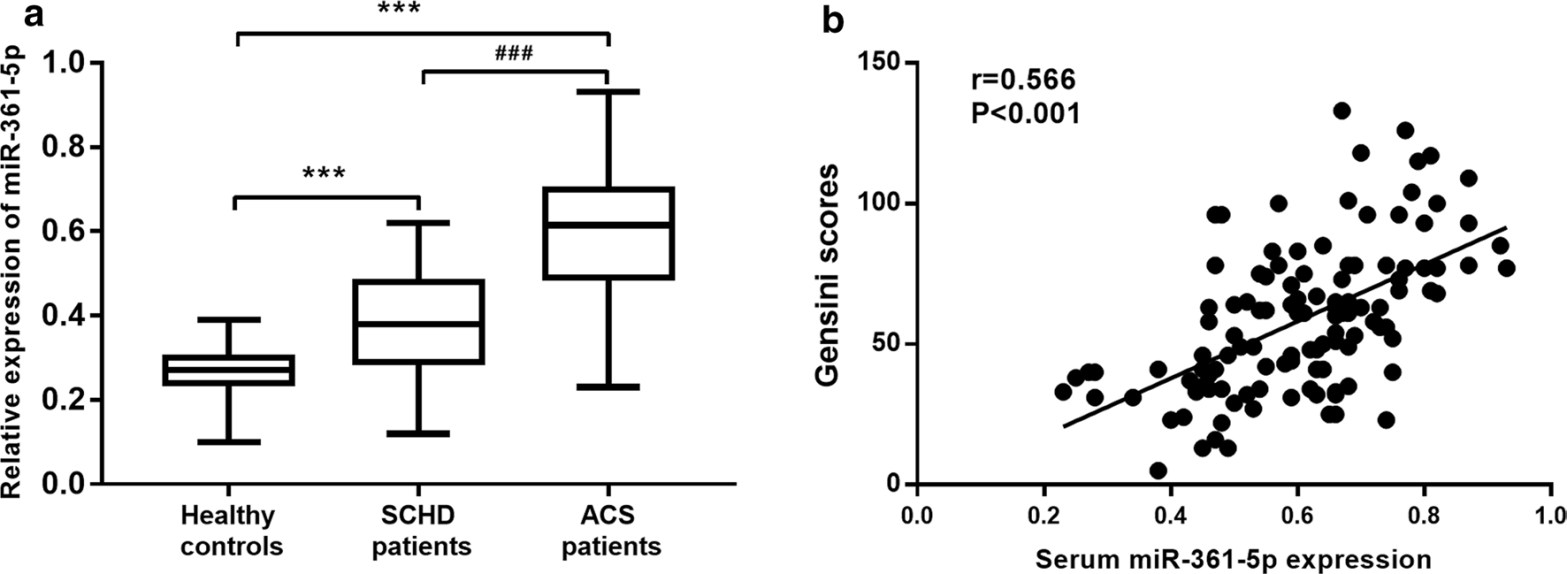
Expression of miR-361-5p and its correlation with Gensini scores in ACS patients. **a** Expression of miR-361-5p in healthy controls, SCHD patients and ACS patients. **b** Correlation of serum miR-361-5p expression with Gensini scores in ACS patients (r = 0.566, *P* < 0.001). (***P < 0.001 vs. Healthy controls, ^###^*P* < 0.001 vs. SCHD patients)

As shown in Fig. 1a, the expression levels of miR-361-5p in healthy controls, SCHD patients and ACS patients were explored. The expression of miR-361-5p was upregulated in SCHD patients and ACS patients compared with that in the healthy controls, and miR-361-5p expression was upregulated in ACS patients compared with SCHD patients (all *P* < 0.001). Additionally, a positive correlation between serum miR-361-5p expression and Gensini score was found in ACS patients (Fig. 1b, r = 0.566, *P* < 0.001).

### Diagnostic potential of miR-361-5p to distinguish ACS patients from SCHD patients and healthy controls

**Fig. 2:**
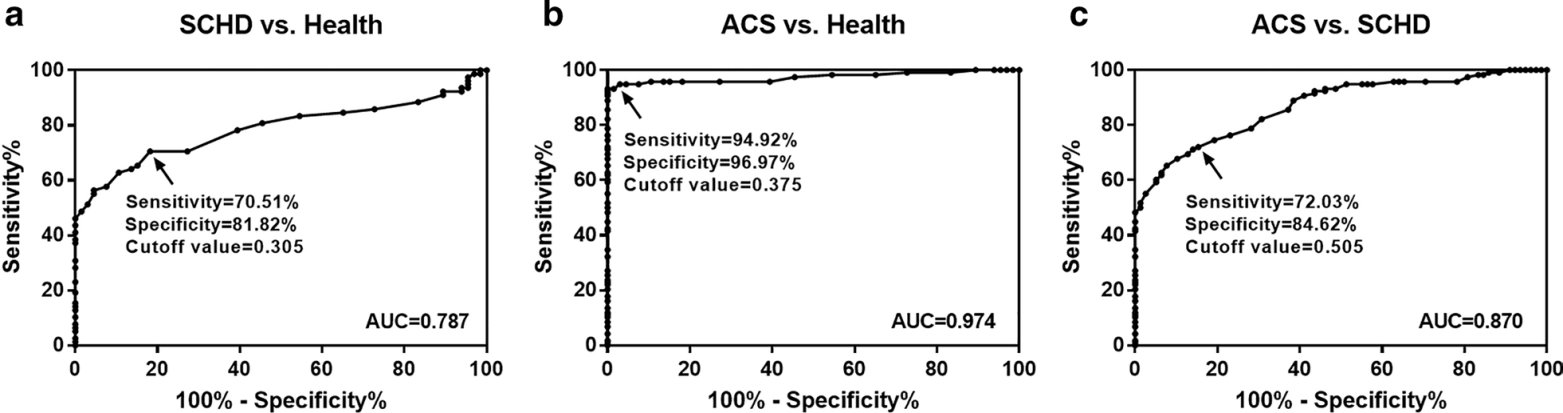
Diagnostic value of miR-361-5p. **a** A ROC curve based on serum miR-361-5p expression in distinguishing SCHD from healthy controls. **b** A ROC curve based on serum miR-361-5p expression for screening ACS from the healthy controls. **c** A ROC curve based on serum miR-361-5p expression for screening ACS from SCHD

To investigate the diagnostic potential of miR-361-5p, ROCs were plotted. As shown in Fig. 2a, the area under the curve (AUC) of miR-361-5p was 0.787, suggesting that serum miR-361-5p has a general diagnostic accuracy in distinguishing SCHD patients from the healthy controls. From Fig. 2b, the AUC of miR-361-5p was 0.974 with a sensitivity of 94.92% and a specificity of 96.97%, indicating that miR-361-5p is of high diagnostic value for screening ACS patients from healthy controls. As can be seen from Fig. 2c, the AUC of miR-361-5p was 0.870 with a sensitivity of 72.03% and a specificity of 84.62%, suggesting that serum miR-361-5p has a certain diagnostic value for screening ACS patients from SCHD patients.

### Association of miR-361-5p with short-term prognosis in ACS patients

**Fig. 3:**
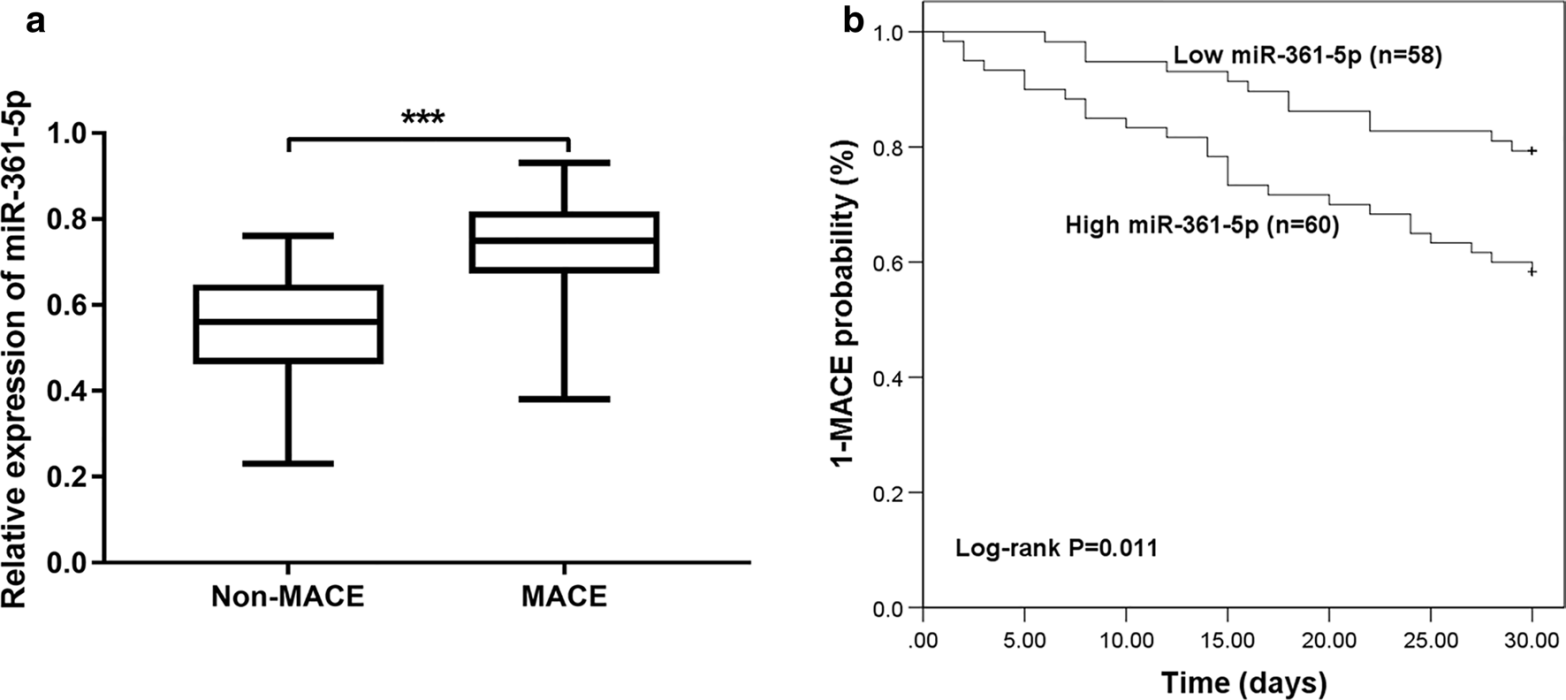
Association of miR-361-5p expression with short-term prognosis of ACS patients. **a** Expression of miR-361-5p in ACS patients with and without MACE. **b** The results of Kaplan–Meier curves showed that patients with high miR-361-5p levels had a higher probability of developing MACE (Log-Rank *P* = 0.011). (***P < 0.001 vs. non-MACE ACS patients)

The results of the 30-day short-term follow-up showed that 37 of 118 ACS patients (31.4%) developed MACE, including 6 deaths, 8 heart failure, 16 unstable angina, and 7 malignant arrhythmias. As shown in Fig. 3a, ACS patients with MACE had significantly higher expression of miR-361-5p than the patients without MACE (*P* < 0.001). According to the median value of miR-361-5p, ACS patients were divided into high and low miR-361-5p expression groups. The results of Kaplan–Meier curves (Fig. 3b) showed that patients with high levels of miR-361-5p had a higher probability of developing MACE (log-rank *P* = 0.011). Further logistic multivariate regression analysis showed that miR-361-5p is an independent risk factor for MACE occurrence in ACS patients and may have the ability to predict the occurrence of MACE (*P* < 0.05, Table 2).

**Table 2:** Multivariate logistics analysis for miR-361-5p in ACS patients

Features	Multivariate logistics analysis		
	OR	95% CI	*P* value
Age	1.228	0.792–1.761	0.742
Gender	1.122	0.807–1.851	0.856
BMI	1.608	0.897–2.341	0.224
TC	1.841	0.908–2.622	0.168
TG	1.908	0.966–3.121	0.077
HDL-C	1.934	0.971–3.257	0.065
LDL-C	2.019	0.982–3.345	0.057
Hypertension	1.755	0.928–2.626	0.234
Diabetes	1.798	0.951–2.974	0.097
WBC	1.713	0.913–2.596	0.276
cTnI	1.858	1.098–2.801	0.047*
Gensini score	2.225	1.749–2.917	0.018*
Drug therapy			
Antiplatelet therapy	1.371	0.785–2.117	0.457
Nitrates	1.108	0.804–1.598	0.895
ACEI/ARB	1.152	0.776–1.853	0.524
Statins	1.214	0.781–1.779	0.661
β-blockers	1.089	0.628–1.597	0.735
miR-361-5p	2.485	1.999–3.208	0.012*

*miR-361-5p* microRNA-361-5p, *ACS* acute coronary syndrome, *OR* odds ratio, *CI* confidence interval, *BMI* body mass index, *TC* total cholesterol, *TG* triglyceride, *HDL-C* high-density lipoprotein cholesterol, *LDL-C* low-density lipoprotein cholesterol, *WBC* white blood cell, *cTnI* cardiac troponin I, *ACEI* angiotensin-converting enzyme inhibitor, *ARB* angiotensin receptor blocker

*P < 0.05

### Correlation of serum miR-361-5p with ED in ACS patients

**Fig. 4:**
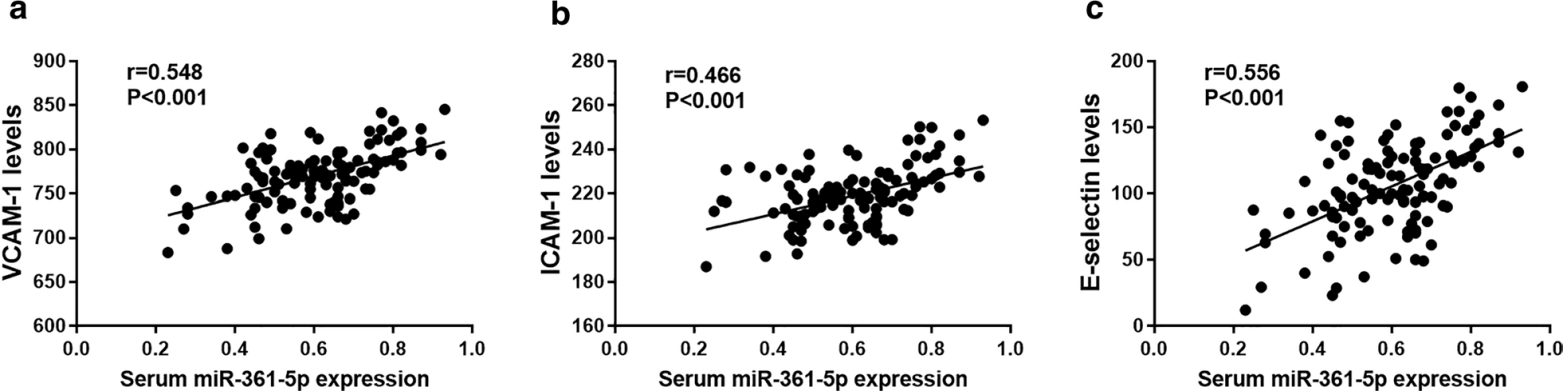
Correlation of serum miR-361-5p expression with ED in ACS patients. **a** Correlation of miR-361-5p expression with levels of VCAM-1 (r = 0.548, *P* < 0.001). **b** Correlation of miR-361-5p expression with levels of ICAM-1 (r = 0.466, *P* < 0.001). **c** Correlation of miR-361-5p expression with levels of E-selectin. (r = 0.556, *P* < 0.001)

From the Fig. 4, the correlation of serum miR-361-5p expression with ED markers in ACS patients was investigated. The results indicated that in ACS patients, serum miR-361-5p expression was positively correlated with the levels of VCAM-1 (Fig. 4a, r = 0.548, *P* < 0.001), ICAM-1(Fig. 4b, r = 0.466, *P* < 0.001), E-selectin (Fig. 4c, r = 0.556, *P* < 0.001).

## Discussion

Numerous studies have reported the crucial roles of miRNAs in the initiation and progression of various human diseases such as cancer, neurodegenerative diseases and cardiovascular diseases [14]. MiRNAs have been found to function in the most important humans' adaptive processes during acute coronary syndrome and myocardial infarction. For example, miRNAs were implied in different apoptotic and inflammatory pathways that could cause higher rate of MACE [15]. Notably, multiple molecular and cellular effectors of the process of MACE development are marked at level of peri-coronary adipose tissue of patients with AMI [16]. Besides, a study has identified miR-33 as an activator of sirtuin 1 and molecular factors implied in coronary thrombus burden of hyperglycemic STEMI patients [17], and the mechanism of intra coronary thrombus is a major factor to cause AMI with ST elevation. Moreover, it has been reported the importance of miRNAs and cardiosomal miRNAs in the post-infarction myofibroblast phenoconversion [18], as well as acting as mediators of post-ischemic myofibroblast activation in vitro and ex vivo models [19]. Furthermore, some functional miRNAs have been found to be associated with ACS, such as miR-21 [20] and miR-146a [21], suggesting that abnormal miRNA expression plays important role in the progression of ACS. In this study, the clinical data of 118 ACS patients, 78 SCHD patients and 66 healthy controls subjects admitted to hospital between 2018 and 2019 were analyzed. All participants met the study criteria and agreed to participate in this study. In addition, there were no studies with clinical data from healthy individuals who did not consent to participate in this study were not studied. The results of this study showed that the expression of miR-361-5p was upregulated in SCHD patients and ACS patients compared with that in healthy controls, and miR-361-5p expression was upregulated in ACS patients compared with that in SCHD patients. In addition, ACS patients had significantly elevated levels of TC, LDL-C, WBC and cTnI and significantly decreased HDL-C level compared with the healthy controls. The data from this study were consistent with previous studies. For example, a significant increase in miR-361-5p expression was also found in CHD patients compared with that in high-risk controls [22]. Wang et al. found that miR-361-5p was significantly increased in AMI disease patients compared to that in healthy volunteers [23]. Moreover, abnormal expression of miR-361-5p has also been found in other diseases. For instance, Ma et al. found that miR-361-5p was downregulated in colorectal carcinoma and gastric cancer compared with the controls [24]. Upregulated miR-361-5p was found in the livers of two obese mouse models and patients with non-alcoholic fatty liver disease [25]. Importantly, a positive correlation between serum miR-361-5p expression and Gensini score was found in the ACS patients, suggesting that miR-361-5p expression is positively correlated with the severity of ACS. Therefore, it is indicated that miR-361-5p might be involved in the progression of ACS.

MiRNAs are considered ideal candidate biomarkers for various human diseases, including ACS. For example, serum miR-501-3p served as a potential biomarker associated with the progression of Alzheimer’s disease [26]. Serum miR-21 and miR-221 were used as potential biomarkers for cerebrovascular disease [27]. In addition, miR-941 might be a potential biomarker for ACS [28]. Thus, the current study explored whether serum miR-361-5p could serve as a potential biomarker for ACS patients. ROC analysis indicated that miR-361-5p had a certain diagnostic value for screening ACS in healthy controls and SCHD patients. Following that, the ability of miR-361-5p to predict the occurrence of MACE was explored. First of all, ACS patients with MACE had significantly higher miR-361-5p expression levels than the patients without MACE. Then, the results of Kaplan–Meier curves indicated that patients with high miR-361-5p levels had a higher probability of MACE than the patients with low miR-361-5p levels. Finally, further logistic analysis showed that miR-361-5p was an independent prognostic factor for predicting the occurrence of MACE in ACS patients. In addition, many evidences have indicated that miR-361-5p may serve as a potential biomarker for other diseases. For instance, a study by Liu et al. showed that miR-361-5p was a novel diagnostic biomarker for glioma [29]. And miR-361-5p expression was an independent predictor of better prognosis in breast cancer [30]. Jin et al. found that miR-361-5p may be a candidate biomarker for early diagnosis [31] and prognosis [32] of non-small cell lung cancer. Thus, it is suggested that serum miR-361-5p may be a useful clinical tool for diagnosing ACS and predicting the occurrence of MACE.

The process of vascular atherosclerotic which underlines coronary damage, and its clinical expressions have multiple pathophysiological bases. In particular, the mechanisms underlying the atherosclerotic damage [33–36] are numerous, and should all be taken advantage of by epigenetic studies. In addition, ED is known to run through the whole process of atherosclerosis, as well as the occurrence and development of CHD. Therefore, exploring the correlation between endothelial function related molecules and ACS disease progression can provide more potential biomarkers and therapeutic targets for ACS disease. Moreover, a large number of miRNAs have been reported to regulate endothelial cell biological function related to the initiation and progression of various disease, including CHD. For example, miR-223-3p could alleviate vascular endothelial injury in kawasaki disease [37]. NEAT1/miR-140-3p/MAPK1 mediated the viability and survival of coronary endothelial cells and affected coronary atherosclerotic heart disease [38]. MiR-17 regulated the proliferation and apoptosis of endothelial cells in CHD [39]. In this study, serum miR-361-5p expression was positively correlated with the levels of ED markers in ACS patients, suggesting that miR-361-5p is closely correlated with ED in ACS patients. Previous studies have found that miR-361-5p is associated with the endothelial function. For instance, a study has found that miR-361-5p can inhibit the viability, migration and tube formation of endothelial progenitor cells [40]. Early study reported that miR-361-5p has anti angiogenic effects through regulating endothelial cell activity [8]. In addition, miR-361-5p could be associated with endothelial cell activity and function, which was closely related to the occurrence and development of CHD by regulating VEGF [9]. Cui et al. found that miR-361-5p inhibited hepatocellular carcinoma cell proliferation and invasion by targeting vascular endothelial growth factor A (VEGFA) [41]. Therefore, miR-361-5p might regulate endothelial cell activity and function in ACS patients by regulating VEGF. However, whether this mechanism can be used for ACS progression remains unclear, and the identification of this mechanism needed further investigation.

Thus, a limitation of this study is that no further mechanistic analysis was performed, which is the focus of future researches. Further investigations are needed to further uncover the molecular mechanisms by which miR-361-5p participation in the physiological and pathological processes of ACS. Another limitation is that the study population is too little, and the number of controls was smaller than the number of ACS patients and even approached half that of ACS patients. Therefore, the clinical value of miR-361-5p needs to be confirmed in a larger study population as well as a control population in further studies.

## Conclusion

In summary, this study indicated that miR-361-5p expression is significantly increased in ACS patients. Aberrant expression of miR-361-5p in ACS patients may be a candidate biomarker for screening ACS and the prediction of MACE.

## Data Availability

The datasets used and/or analysed during the current study are available from the corresponding author on reasonable request.

## Abbreviations

miRNAs: MicroRNAs
miR-361-5p: MicroRNA-361-5p
CHD: Coronary heart disease
SCHD: Stable coronary heart disease
ACS: Acute coronary syndrome
AMI: Acute myocardial infarction
UA: Angina pectoris
ED: Endothelial dysfunction
VCAM-1: Vascular cell adhesion molecule 1
ICAM-1: Intercellular adhesion molecule 1
3′-UTR: 3′-Untranslated region
VEGF: Vascular endothelial growth factor
cTnI: Cardiac troponin I
CK-MB: Creatine kinase MB
ECG: Electrocardiogram
NSTEMI: Non-ST-segment elevation myocardial infarction
STEMI: ST-segment elevation myocardial infarction
BMI: Body mass index
TC: Total cholesterol
TG: Triglyceride
HDL-C: High-density lipoprotein cholesterol
LDL-C: Low-density lipoprotein cholesterol
WBC: White blood cell
MACE: Major adverse cardiac events
qRT-PCR: Quantitative real-time PCR
SD: Standard deviation
ROC: Receiver operating characteristic
AUC: Area under the curve
VEGFA: Vascular endothelial growth factor A
